# Be Reasonable

**Published:** 1878-06-20

**Authors:** 


### BE REASONABLE.

If a dun should, by mistake, be put into the journal of one who has paid, let him not fret. It is not surprising that such things occur occasionally in addressing large numbers. And to those who have not paid, we again say, do so at once. Don't wait for bills to be sent. It costs the editors much labor and expense to send out these bills.

